# RegentK and Physiotherapy—Electrodermal Mapping

**DOI:** 10.3390/medicines1010022

**Published:** 2014-09-30

**Authors:** Gerhard Litscher, Daniela Litscher, Michael Ofner, Ingrid Gaischek, Daniela-Eugenia Malliga

**Affiliations:** 1 Research Unit for Complementary and Integrative Laser Medicine, Research Unit of Biomedical Engineering in Anesthesia and Intensive Care Medicine, and TCM Research Center Graz, Medical University of Graz, Auenbruggerplatz 29, 8036 Graz, Austria; E-Mails: daniela.litscher@medunigraz.at (D.L.); ingrid.gaischek@medunigraz.at (I.G.); 2 Department of Sports Physiology, University of Vienna, Auf der Schmelz 6, 1150 Vienna, Austria; E-Mail: michael.ofner@medyco.net; 3 Division of Cardiac Surgery, Department of Surgery, Medical University of Graz, Auenbruggerplatz 29, 8036 Graz, Austria; E-Mail: daniela-eugenia.martin@medunigraz.at

**Keywords:** RegentK, Khalifa therapy, physiotherapy, anterior cruciate ligament, electrical skin resistance, electrodermal mapping

## Abstract

**Background:**

Within this study, a new system which measures and analyzes electrical skin impedance in 48 channels within a 2.5 × 3.5 cm matrix is used in rehabilitation medicine for the first time.

**Methods:**

Electrodermal activity was measured in 20 patients before and after two different non-surgical treatments of a completely ruptured anterior cruciate ligament. The first treatment, RegentK, was developed by Mohamed Khalifa, the second is a standard physiotherapy.

**Results:**

The patients in the two groups were age-matched, and all demographic data showed no significant differences. It was interesting that electrodermal activity was significantly decreased only after RegentK.

**Conclusion:**

We conclude that not only local effects of pressure application are responsible for these results, rather as yet unknown neurovegetative mechanisms have to be taken into consideration.

## 1. Introduction 

Sports injuries are common; within a ranking with respect to cause of injury they are in second place with 21% [1]. Especially, knee lesions very often lead to long-term disabilities [2]. Twenty percent of sport-related knee injuries involve the anterior cruciate ligament (ACL) [3].

New studies show that conservative treatments like RegentK therapy (developed by Mohamed Khalifa in Hallein, Austria) can improve stability, rehabilitation, and outcome tremendously [4,5,6]. Therefore, research should give full attention to this non-invasive treatment method, especially in comparison to surgical techniques. Following RegentK, knee stability can be restored in a very short time and athletes can return to sports and an active lifestyle very soon [4,5,6]. This was shown in many patients, among them several top athletes, over the last few decades [7,8].

Electrodermal mapping is a new method developed partly at the Medical University of Graz four years ago [9,10]. It allows a highly precise electrical characterization of different areas of the skin for the first time [9,10].

In this randomized controlled study we investigated acute effects of RegentK compared to standard physiotherapy on electrical skin properties of knee tissues in patients with completely ruptured ACL using new modern technical instruments.

## 2. Materials and Methods

### 2.1. Patients

Two patient groups were investigated. Ten patients were assigned to group A and received RegentK, and ten patients were assigned to group B and received physiotherapy. The most important demographical data of the two patient groups can be seen in Table 1.

**Table 1:** Demographical data of both patient groups. Data are given as means ± SD.

	Group A (RegentK)	Group B (Physiotherapy)
age (years)	31.3 ± 8.5	34.8 ± 10.2
range (years)	20–43	19–47
sex	8 f, 2 m	6 f, 4 m
cause of injury (no. of patients)	skiing (8), household (1), volleyball (1)	skiing (9), volleyball (1)
injured side (no. of patients)	left (5), right (5)	left (4), right (6)

Inclusion and exclusion criteria can be found in Table 2.

**Table 2:** Inclusion and exclusion criteria for this study.

Inclusion Criteria	Exclusion Criteria
- unilateral complete rupture of the ACL (less than 14 days ago), verified by magnetic resonance imaging - age: 18–49 years - normal body weight: BMI (body mass index) 18–25 - regular exercise level - knee instability: experienced at least one giving-way - dysfunction: knee range-of-motion: reduced or inhibited	- preceding surgical intervention (including arthroscopy) on the injured knee - metabolic disorders like diabetes mellitus - autoimmune diseases

As far as the study design allowed, patients were informed about the investigation. The study was approved by the ethics committee of the University of Salzburg, Austria (ID-no. 21-232 11-12 sbg + amendment), and registered at clinicaltrials.gov under the ID-no. NCT01762371. All participants provided written informed consent.

### 2.2. RegentK

In a previous paper [5], it was outlined that “Khalifa therapy is described as functional-pathological [7]. In this approach, function is the primary concern, not anatomy. The most important thing is not the ruptured ligament itself, but its function/dysfunction. Khalifa therapy restores the function of the knee in a natural way. During the 60–90 min of his manual therapy, he applies pressure to the injured knee in order to activate the self-healing processes of the human body, using his hands as an instrument for both measurement and therapy. Over periods of varying length, he applies increasing pressure on a spot before moving on to the next spot. The frequency of pressure application depends on the patient’s physiological reaction. The force of the pressure is not comparable to that normally used in acupressure in Traditional Chinese Medicine [11]; it is much higher. Mohamed Khalifa’s method is based on manual pressure of varying frequency and does not damage the body, but supports it in its own natural healing activities. If one cuts through an elastic band and sews it together again, one cannot expect it to be as elastic at the stitching point as it was before. It is the same with human ligaments, and if the elasticity is disrupted anywhere in the human body, the whole system is affected” [7,8].

### 2.3. Physiotherapy

During physiotherapeutic intervention, the focus was put on the myofascial structures of tissues surrounding the knee joint; the upper part of the body and the upper extremities were not included in the treatment. Two basic techniques were used during the control intervention: (a) a static manual treatment, *i.e.*, pressing trigger points usually to be found in the muscle belly (typically, e.g., in the m. quadriceps, the m. biceps femoris, m. popliteus, m. gastrocnemius); and (b) static-dynamic stretching intermuscular techniques in the areas of the tractus iliotibialis, the medial femoral muscles directed toward the knee or between (and in) the muscle bellies of the m. gastrocnemius.

Both techniques were performed at the patient’s individual pain threshold, and static trigger points were pressed 3–5 times (for a maximum of 30 s each) until the tension and pain subsided. The myofascial longitudinal and crosswise stretchings were also repeated 3–5 times, slowly and dynamically.

Before and during the treatment (duration about 60 min), patients were informed about potential physiologic reactions of the interventions [12,13,14,15,16].

### 2.4. Evaluation Parameters

**Figure 1:**
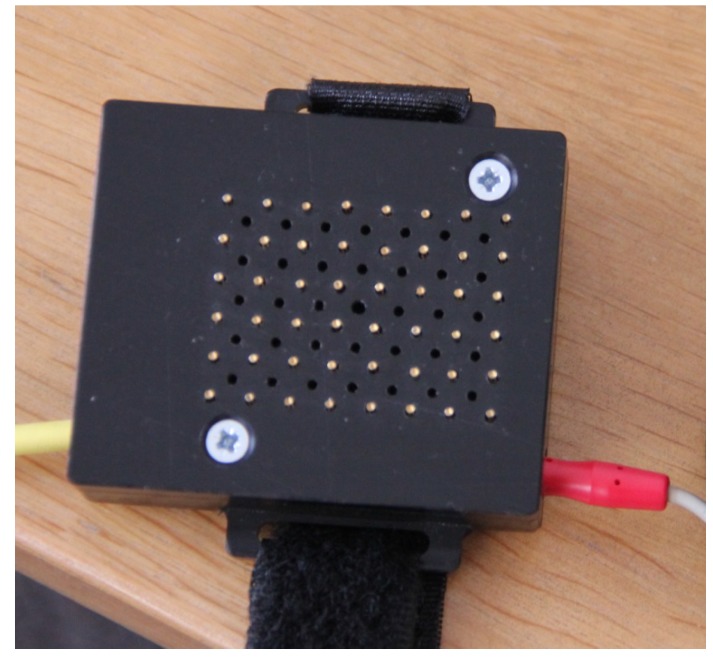
Sensor part of the measurement system for electrodermal mapping.

**Figure 2:**
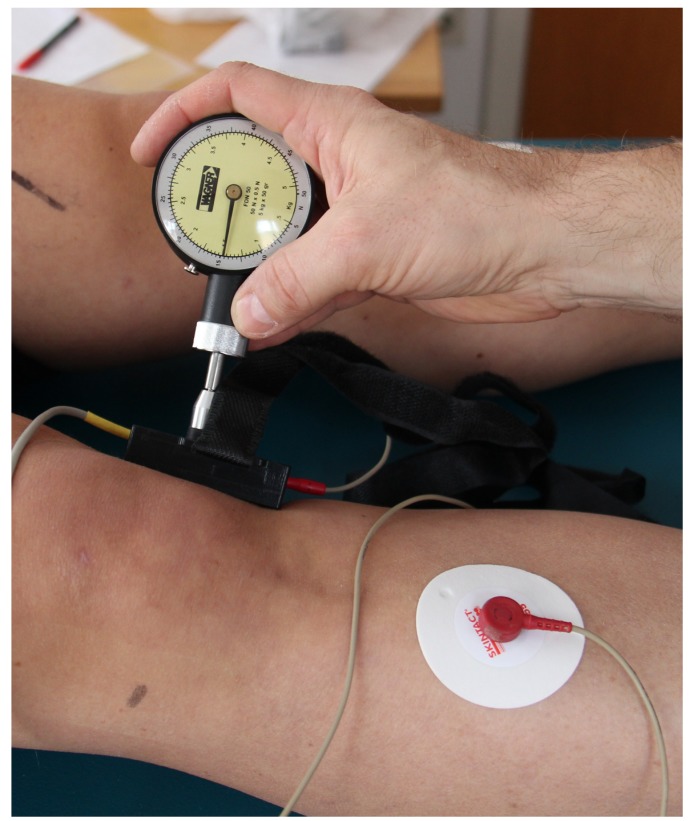
Application of the GEDIS system with a contact pressure of 15 N. The grounding electrode can be seen on the right.

The new system called GEDIS (Graz ElectroDermal Impedance measurement System) [9,10] was developed to measure skin impedance at different locations on the human body. It allows single short measurements, but also continuous monitoring over hours. It is an 8 × 6 electrode array with spring-mounted electrodes. The measurement circuit is time multiplexed across the 48 channels (Figure 1). The electrodes have a diameter of 0.9 mm and the material is gold-plated beryllium copper. A calibration against a standard is not possible because there is no standard device available at the moment. It is not possible to measure the pressure under each probe while monitoring skin resistance. However, we applied the electrode matrix with a controlled contact pressure of 15 N (Force Dial FDN 50, Wagner Instruments, Greenwich, CT, USA) (see Figure 2). Registrations were carried out using an electrical current of 1.46 µA [9,10].

### 2.5. Procedure

The skin was cleaned with alcohol in the area where registration was to take place, and all metallic and/or conductive items were removed from the body. 

All patients were investigated in a supine position under similar conditions. Electrodermal registrations were performed at three sites on the knee: lateral, medial, and proximal above the patella. As control, the same spots were also tested on the healthy knee (this knee did not receive any therapy or manual manipulation).

### 2.6. Statistical Analysis

For each measurement, the mean of all 48 registration sites was calculated. These mean values of electrical skin resistance on both legs were tested with paired t-test (before *vs.* after; SigmaPlot 12.0, Systat Software Inc., Chicago, IL, USA). The level of significance was defined as *p* < 0.05.

## 3. Results and Discussion 

All recordings were reliable at all 48 skin sites. The initial conditions (knee instability and dysfunction), as well as the mean age and other patient data, showed no significant differences between group A and group B (see Table 1). All 20 patients completed the study procedure.

**Figure 3:**
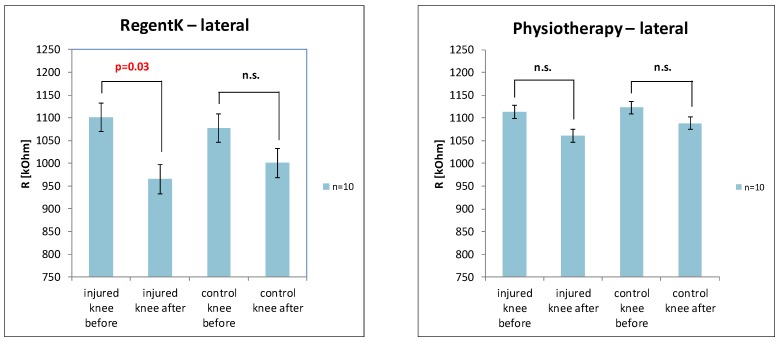
Graphical presentation of the changes in electrodermal activity on the lateral measurement position of the injured and control knee after RegentK (**left**) and physiotherapy (**right**).

Figure 3 shows a significant decrease of skin resistance after RegentK on the lateral side of the injured knee. In contrast, physiotherapy did not show significant decreases, however, the trend was the same.

**Figure 4:**
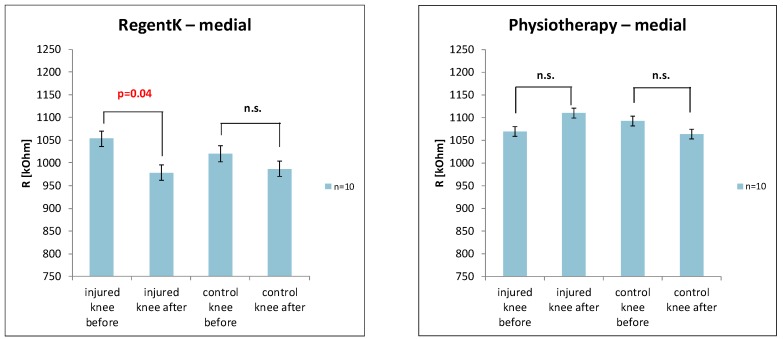
Changes in skin resistance on the medial side of the knee ((**left**) RegentK; (**right**) physiotherapy).

The electrodermal activity values on the medial side of the knee are presented in Figure 4. Similar results were found with RegentK (significant decrease on the injured side).

**Figure 5:**
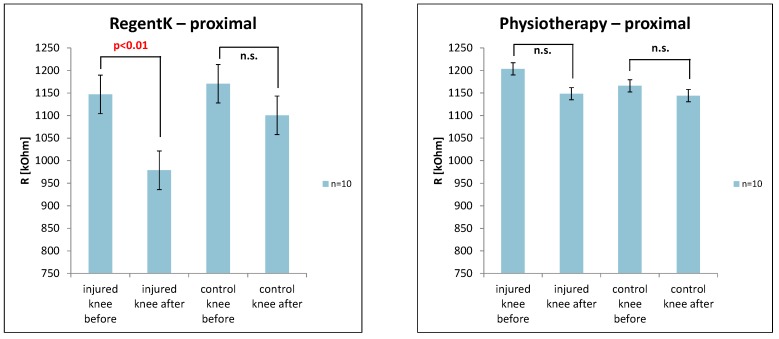
Changes in electrodermal activity on the frontal site.

The next figure (Figure 5) shows the values of the electrodermal activity on the measurement site proximal above the patella. Again, RegentK induced a significant decrease on the injured knee.

Electrodermal activity changes could be observed on all three measurement points on the injured knee after RegentK. This was not the case after physiotherapy. It should also be mentioned that within this randomized controlled study, the function of all ten knees with a ruptured ACL was restored after RegentK, but not after physiotherapy. All patients in group A were able to bend their knees immediately after the end of RegentK. In group B, some success could also be seen, but not to the same extent as in group A.

In preliminary RegentK studies, changes in regional oxygen saturation [5] and temperature [6] could be found. This article describes changes in electrodermal activity in patients with completely ruptured ACL for the first time, using a new technology. There are no data available at the moment concerning electrodermal activity in the area of the knee (see PubMed). However, there are some trials and new approaches for detection of leg movement using segmental electrical impedance changes [17]. This study showed that lower leg movement could be easily measured by the impedance measurement system with two pairs of skin electrodes. The goal of our study was different; we wanted to find out whether the different kinds of therapy induced different changes in electrodermal activity in the area of the knee. This could be detected with the new system described in this study. It is interesting that other studies demonstrated that temperature in the same area also changed significantly, as described by our research team in a previous study [6]. It seems that changes of the neurovegetative state play a major role in RegentK therapy.

The previously existing limitations of electrodermal skin resistance measurements were the measurement area (point selectors with a tip only, representing a hand-held 1-channel system) and related problems (pressure, angle) and also too few registration sites (in most cases only punctual measurements, no multi-channel systems) [10,18,19]. In contrast to our new electrode configuration (electrode diameter 0.9 mm), the electrodes of the system of Colbert *et al.* [19] have a diameter of 4 mm and are fixed separately at the body surface with an elastic adhesive or cloth wrist band. “Confounding factors, such as skin moisture, electrode pressure, stratum corneum thickness, electrode polarization and other factors, have led many to assert that the reportedly distinct electrical characteristics are attributable to external factors and/or artifacts” [20]. Our system allows, for the first time, simultaneous and continuous, multi-channel measurements [10].

It should be mentioned here that the group of patients treated with RegentK includes more females and is a bit younger, that could be one of the reasons for the higher response of both knees, given their higher blood activity.

Two different techniques have been applied to the physiotherapy group, static manual and static-dynamic stretching. This combination could be a further reason for the lower response of these patients to the treatment, maybe focusing on one of them or looking for an optimal combination would give different results. 

As mentioned before, the GEDIS system has not been calibrated due to the unavailability of a standard object. This makes the response of the system unknown, so it should also be mentioned that the reduced response of the physiotherapy group may just be a matter of thresholds.

## 4. Conclusions 

In conclusion, electrodermal mapping is a method, which allows calculation of electrodermal activity with greater accuracy than in the past. The method was used for the first time in rehabilitation medicine to obtain quantitative and qualitative bioelectrical values. Electrodermal activity was significantly decreased after RegentK. Therefore we conclude that not only local effects of pressure application have to be taken into account, but also as yet unknown neurovegetative mechanisms. Further studies are absolutely necessary to confirm or refute our preliminary results on this topic.
